# Cilia‐related diseases

**DOI:** 10.1111/jcmm.17990

**Published:** 2023-10-13

**Authors:** Zhanhong Ren, Xiaoxiao Mao, Siqi Wang, Xin Wang

**Affiliations:** ^1^ Hubei Key Laboratory of Diabetes and Angiopathy Medicine Research Institute, Xianning Medical College, Hubei University of Science and Technology Xianning P. R. China; ^2^ School of Basic Medical Sciences Xianning Medical College, Hubei University of Science and Technology Xianning P. R. China; ^3^ School of Mathematics and Statistics Hubei University of Science and Technology Xianning P. R. China

**Keywords:** cilia, ciliopathy, motile cilia, primary cilia, primary ciliary dyskinesia

## Abstract

More and more attention is paid to diseases such as internal transfer and brain malformation which are caused by the abnormal morphogenesis of cilia. These cilia‐related diseases are divided into two categories: ciliopathy resulting from defects of primary cilia and primary ciliary dyskinesia (PCD) caused by functional dysregulation of motile cilia. Cilia are widely distributed, and their related diseases can cover many human organs and tissues. Recent studies prove that primary cilia play a key role in maintaining homeostasis in the cardiovascular system. However, molecular mechanisms of cilia‐related diseases remain elusive. Here, we reviewed recent research progresses on characteristics, molecular mechanisms and treatment methods of ciliopathy and PCD. Our review is beneficial to the further research on the pathogenesis and treatment strategies of cilia‐related diseases.

## 1 INTRODUCTION

**FIGURE 1:**
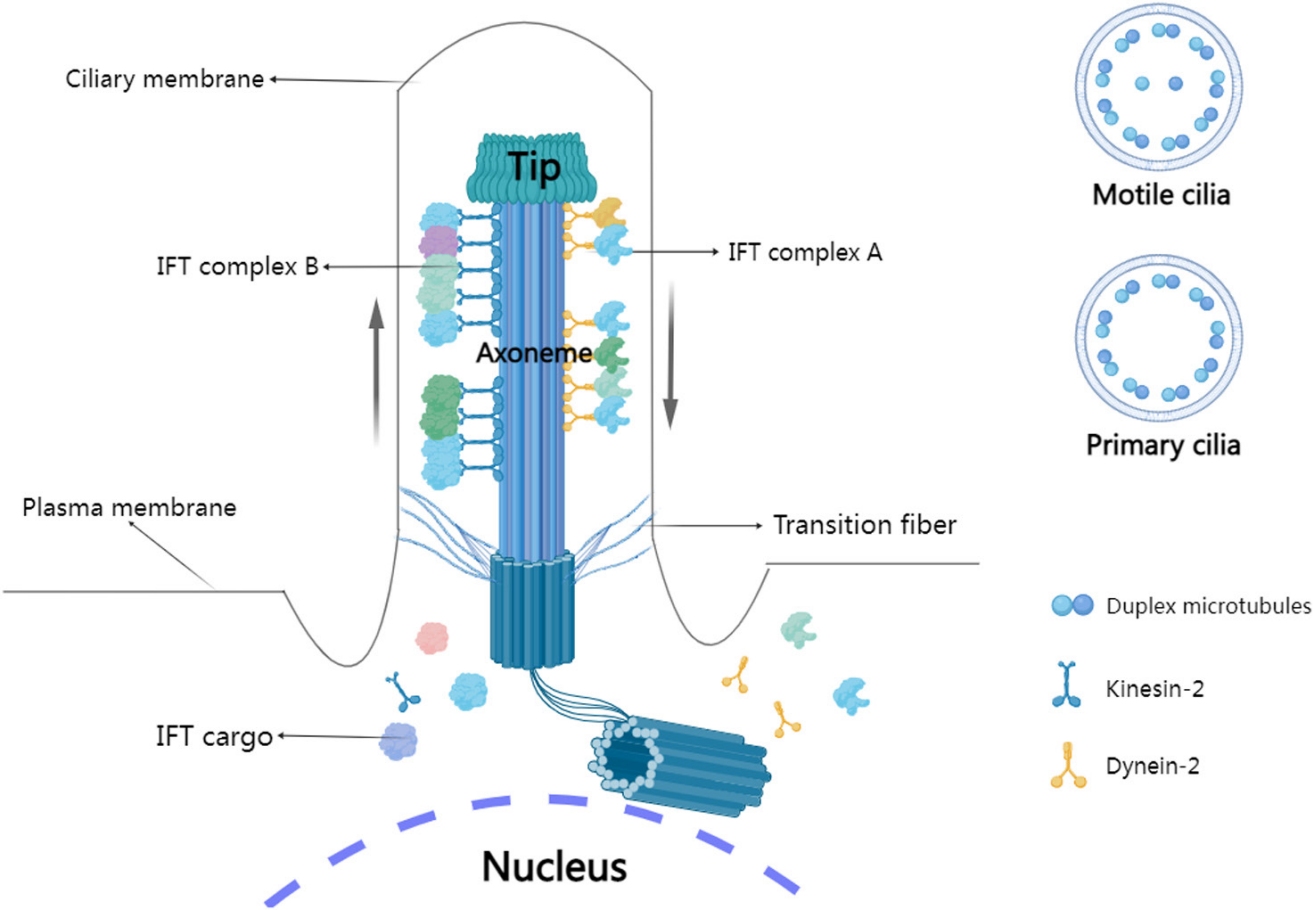
Schematic diagram of the IFT.

Cilia‐related diseases are caused by structural and functional disorders of cilia on cells, affecting multiple organ systems in the human body.^1^ Cilia are highly conserved organelles that project from the cell surface and are divided into primary cilia and motile cilia. The primary cilia are stationary and called ‘cell receptors’ which can sense and transduce extracellular signals. The function of motile cilia is mainly to assist the movement of cells or to affect the fluid environment around the cell. The major structural difference is that the motile cilia are ‘9 + 2’ groups of axial microtubules while the primary cilia are ‘9 + 0’ (Figure 1). Ciliary assembly and maintenance depend on the intraflagellar transport (IFT). The IFT complex contains more than 20 kinds of IFT proteins which can be divided into IFT‐A and IFT‐B (Figure 1).^2^ Currently, mutations in the IFT genes are known to cause mild or severe cilia‐related diseases.

In addition, other cilia‐related genes such as the ciliary membrane receptor protein genes, sonic Hedgehog (SHH)‐related genes in the ciliary signalling pathway and cilia basal body related genes also play a critical role in ciliary assembly and maintenance.^3^ Mutations in cilia‐related genes can cause ciliary defects including the shorter or longer cilia. It will lead to a variety of cilia‐related diseases.

**FIGURE 2:**
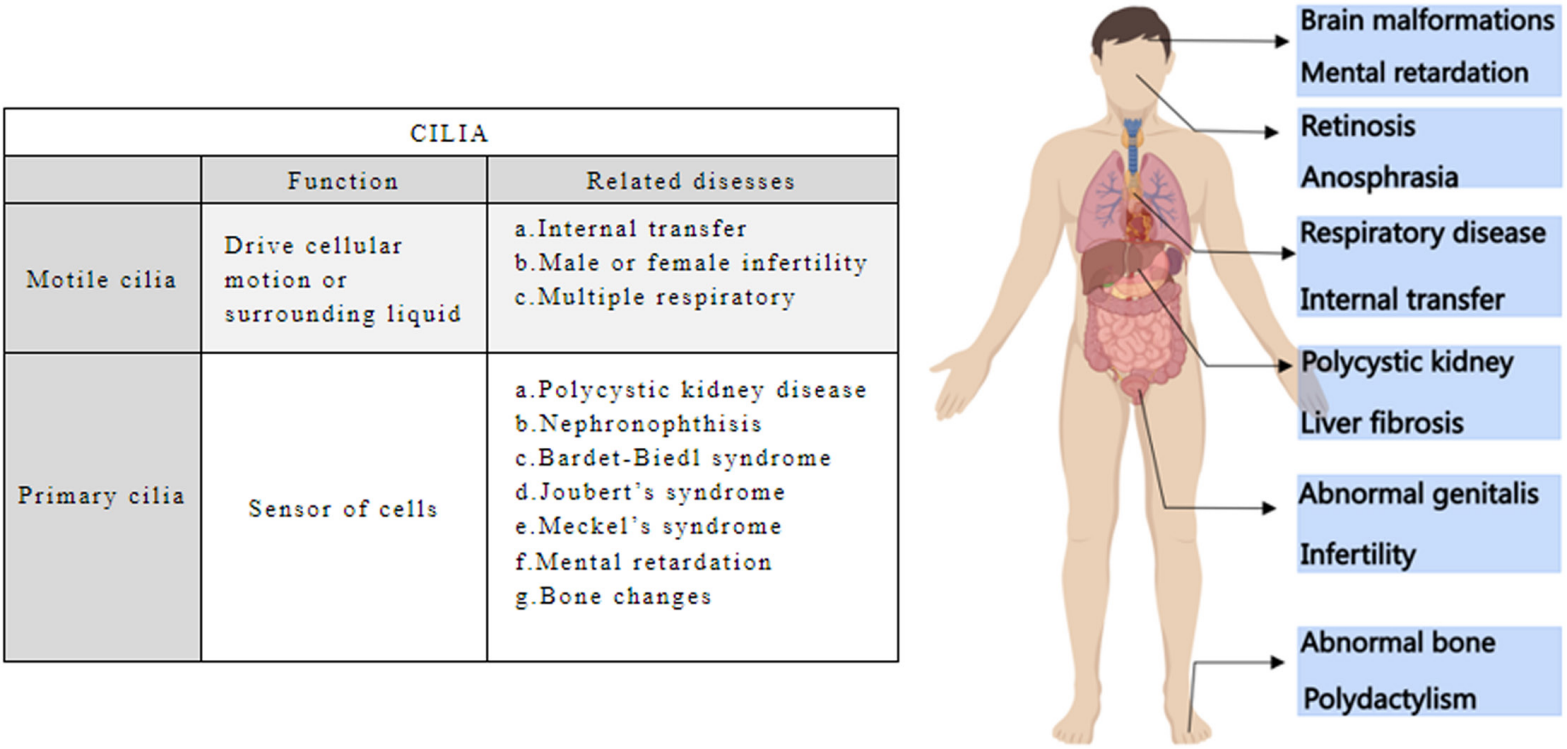
Clinical manifestation of cilia‐related diseases.

Cilia are involved in cellular signalling pathways such as TGF‐β, Wnt and Hedgehog signalling and participate in various cellular processes such as proliferation, differentiation and cell cycle. Cilia are present on almost all cell types in the human body. Because of their wide distribution, ciliary gene mutations or morphological changes can affect a variety of tissues and organ systems (Figure 2). It is called ‘ciliopathy’ that primary cilia dysfunction leads to organs abnormalities.^4^ Besides, ‘primary ciliary dyskinesia (PCD)’ is one major group of motile cilia‐related diseases. Here, we summarize recent research progresses on the pathogenesis of typical cilia‐related diseases.

## 2 PRIMARY CILIA DISORDERS: CILIOPATHY

### 2.1 Polycystic kidney disease

The polycystic kidney disease (PKD) can be divided into autosomal dominant polycystic kidney disease (ADPKD) and autosomal recessive polycystic kidney disease (ARPKD). The most obvious clinical manifestation in patients with PKD is enlarged liver and kidney.

In the United States and Europe, ADPKD is the most common potentially lethal autosomal dominant disease.^3^ It is mostly asymptomatic before onset, but when symptoms appear, it progresses very rapidly. Its common clinical manifestations include haematuria and significant lower waist pain. ADPKD‐related genes are *PKD1* and *PKD2* which encode polycystin‐1 (PC1) and polycystin‐2 (PC2), respectively. The PC1 and PC2 are localized on the primary cilia and form a multiple transmembrane protein complex.^5^ Among patients with ADPKD, the majority of patients have the *PKD1* mutations which accounts for about 80 percent, and it progresses faster in those with *PKD1* mutations.^6^

The gene that causes the ARPKD is *polycystic kidney and hepatic disease 1* (*PKHD1*). There is a synergistic effect between *PKD1* and *PKHD1*, and this effect is associated with a higher lesion rate in ARPKD patients.^7^ The *PKHD1* encodes fibrocystin or polyductin (FPC) which is a single transmembrane protein. It not only localizes on cilia but also in other organelles. The role of FPC in cilia remains unclear. Compared with ADPKD, most of ARPKD patients are newborn babies or young children and 80 percent of children have severe high blood pressure.^8^

It is reported that sirolimus plays an important role in treating animal models with ADPKD.^9^ The effect of ursodeoxycholic acid (UCAD) for treating ARPKD has been discovered.^10^ UCDA inhibited the formation of liver cysts in ARPKD rats. However, the more common treatment for these diseases is kidney surgery.

### 2.2 Bardet–Biedl syndrome

The Bardet–Biedl syndrome (BBS) is one of the most serious ciliopathies. It is an autosomal recessive hereditary disease. Its clinical manifestations include severe retinitis pigmentosa, obesity, hypogonadism and a wide range of additional symptoms. Because obesity is so common among people with BBS, there are also many patients with type 2 diabetes, which may be related to the degree of obesity. Developmental delays and cognitive deficits are also common in BBS, which also lead to movement disorders and learning difficulties in some patients.

At present, there are 21 known pathogenic genes of BBS *(BBS1‐BBS21)*. Mutations in these genes account for 80 percent of clinical diagnoses, the most prominent of these are genes associated with the BBSome complex (Heterooctamer protein complex plays a key role in primary cilia homeostasis).^11^, ^12^ This is the most effective way to detect BBS diseases so that early intervention can be started.

Because the molecular pathogenesis of BBS gene pleiotropy has not been completely studied, there are a few targeted therapies for BBS and symptomatic treatment is applied. At present, setmelanotide for obese patients with BBS is already on the market.^13^

### 2.3 Joubert's syndrome

Joubert's syndrome (JS) is a ciliopathy characterized by midbrain–hindbrain malformation (also known as the molar tooth sign), developmental retardation, dystonia and respiratory disorders. With subsequent studies, due to the disease's involvement of multiple organs, especially the kidneys and liver, it is collectively referred to as Joubert's syndrome and related diseases (JSRD).^14^

To date, more than 40 disease‐causing genes have been identified. All of these genes are involved in encoding cilia or cilia‐related proteins. We have listed a few genotypes (Table 1). Agenesis of the corpus callosum was also found in patients with the *CPLANE1* gene mutation. Gene mutations in *AHI1, TEME67* and *CEP290* are also occasionally found in patients with epilepsy JS. At the same time, JS pathogenic genes also have genetic overlap with many other ciliopathies such as BBS, Meckel syndrome, isolated nephronophthisis and oral‐facial‐digital syndromes.^15^, ^16^

**TABLE 1:** Cilia‐related diseases and their treatments.

Related Gene	Proteins	Treatment	Disease
*PKD1/PKD2* *PKHD1*	Polycystin‐1/polycystin‐2	Sirolimus Ursodeoxycholic acid	Polycystic kidney disease
*BBS1‐21* *ARL16* *MKKS* *MKS1* *WDPCP*	BBS proteins	Setmelanotide (obesity)	Bardet–Biedl syndrome
*CEP290* *AHI1* *TMEM67* *CPLANE1* *TCTN1* *TCTN2* *NPHP3* *RPGRIP1L*	/	Intelligence diagnosis and health monitoring	Joubert's syndrome
*GAS2L2* *CCDC164* *DNAH* *NEK10*	/	Airway clearance techniques Systemic antibiotics	Primary ciliary dyskinesia

*Note*: ‘/’ represents corresponding proteins are unknown.

Due to the complexity of the disease and its prevalence in children, there is currently few targeted treatment. It is suggested that people with JS should test their scale intelligence quotient (FSIQ), the generalized ability index (GAI) and general ability.^17^

### 2.4 Cilia‐related cardiovascular disease

**FIGURE 3:**
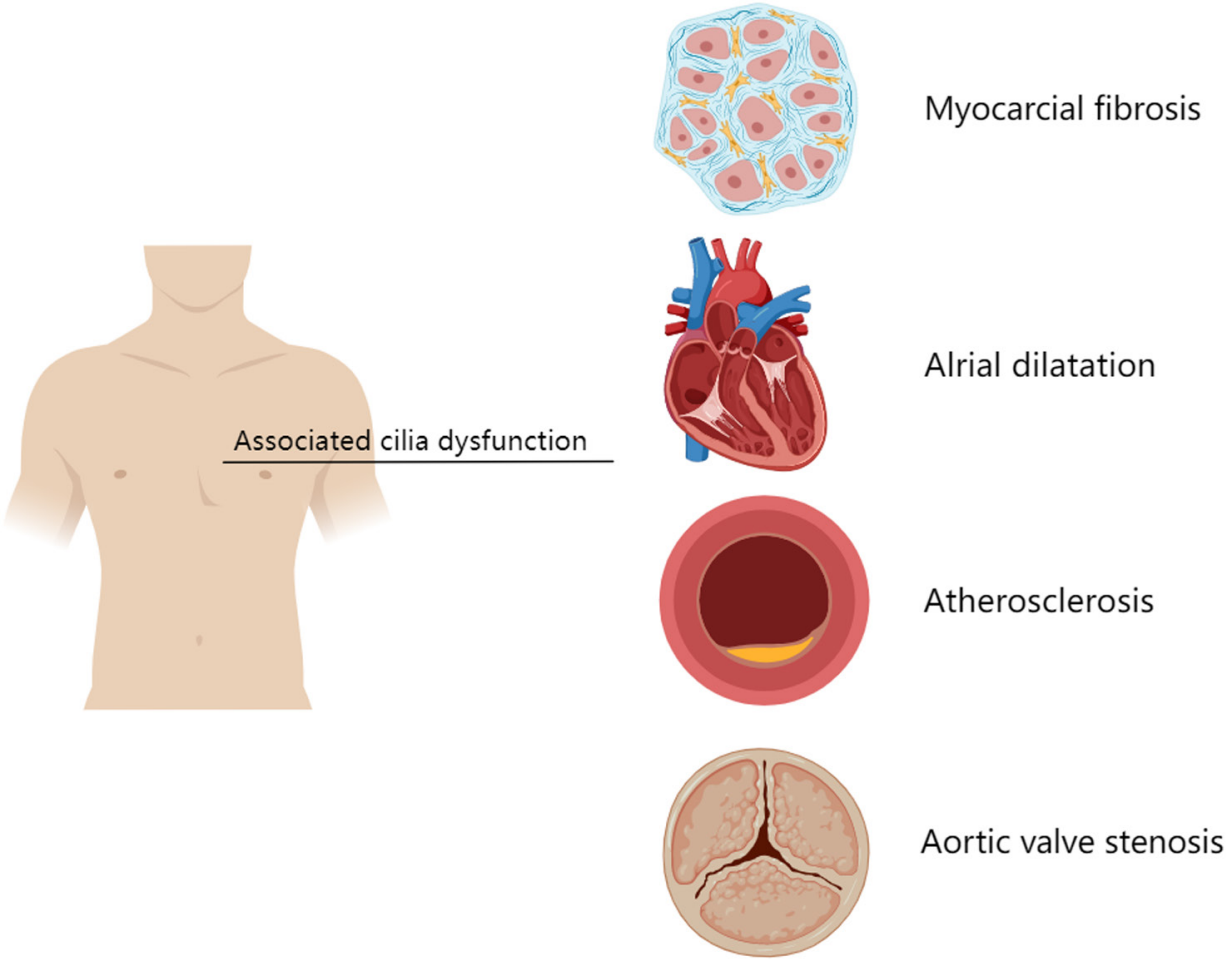
Cilia associated cardiovascular disease.

Numerous studies have also shown that primary cilia play a critical role in congenital or acquired heart disease (Figure 3).^18^, ^19^ Mitral valve prolapse (MVP) can cause arrhythmia, heart failure and sudden cardiac death. Loss of the primary cilia gene during development may lead to severe mitral valve disease.^20^ The congenital aetiology of MVP is characterized by disintegration and fragmentation of collagen and elastin in ECM (extracellular matrix).^21^, ^22^ It has been shown that cilia damage caused by gene mutation of *DZIP1* further develops into functional MVP. MVP is more common in adults, and ciliated mitral disease is more commonly myxomatous.

Another common form is fibrosis of the left ventricle, which can lead to arrhythmias, atrophy of heart muscle cells and heart failure.^23^, ^24^ Cardiac fibroblasts contain primary cilia and their structural proteins, and PC1 are involved in key responses to myocardial fibrogenesis. Together, these organelles are involved in the fibre signalling of transforming growth factor β‐1 (TGF‐β1).^25^, ^26^ In addition to regulating fibrosis, primary cilia also play a vital role in regulating atherosclerosis, ventricular hypertrophy and cardiovascular failure.^27^, ^28^, ^29^

The most common treatment for this disease is surgery, but as its mechanisms are discovered, more beneficial targeted therapies will gradually be found and applied.

## 3 MOTILE CILIA DISORDERS: PRIMARY CILIARY DYSKINESIA

Usually, motile cilia do not occur singly, but multiple ones on the same cell.^30^ They are commonly found in the airway, sinuses,^31^, ^32^ ventricles, ependymal membranes^33^, ^34^ and fallopian tubes.^35^ The set of symptoms caused by motor ciliary dysfunction due to a genetic mutation is called primary ciliary dyskinesia.

Human airway epithelial cells are characterized by motile cilia and cooperate with airway mucus to perform cleaning function.^36^, ^37^ Once the cilia is dysfunctional, the epithelial cell's defence ability will be significantly weakened, which may lead to a series of airway disease. Repeated respiratory infections can develop into more serious and malignant lung diseases including chronic obstructive pulmonary disease (COPD) over time.^38^ The airway disease is extremely common in patients. It includes the nose, upper and lower respiratory tract and middle ear.^39^ Patients with these diseases show symptoms from infancy. The clinical manifestations include chronic persistent nasal congestion, chronic sinusitis, chronic persistent wet cough, recurrent bronchitis or pneumonia, and it almost always develops bronchiectasis eventually.^40^ The characteristics of the disease may vary with age. Due to repeated and chronic airway infections, some patients also have severe lung disease and abnormal lung function as early as infancy.^41^ The common treatment for it is antibiotics and airway clearance therapy. In rare cases, surgical excision or a lung transplant is used. However, it has been shown that cigarette smoke has adverse effects on mucocilia, so it is important to take necessary preventive measures for these diseases, such as avoiding smoke and quitting smoking.

Another common symptom of PCD is infertility. Female infertility is caused by the disturbance of the tubal cilia. At present, its mechanism is not clear. There are some advances in male infertility. Three clinical manifestations of male PCD patients are known and named azoospermia, teratospermia and oligospermia. They result from the dysfunction of motile cilia gene and the power arm disorder or loss.^42^ Different gene mutations also cause different manifestations of the disease. Due to PCD male infertility patients are very few, most data are not yet known especially the specific pathogenic genes.

More than 40 genetic mutations have been identified. The symptoms of PCD usually begin in the neonatal period.^42^ Half of patients with PCD are associated with internal transfer, part of the cause of these patients' disease is the embryonic nodal ciliary dyskinesia, these motile cilia are active only during foetal development, and their movement is rotational, so when they have motor defects, they may cause translocation.^43^

Because most of these patients have severe neutrophil inflammation,^41^ macrolide antibiotics have been shown to have a certain therapeutic effect on these patients. There is few targeted treatment for PCD disease, comprehensive genetic testing, antibiotic treatment and prophylaxis are commonly used.

## 4 CONCLUSION

Presently, more and more studies have been focused on cilia. Based on these research advances, the diagnosis and treatment of clinical patients have been greatly improved. But much more needs to be done to address these complex and diverse diseases, many aspects remain unknown. In particular, the in‐depth understanding of the pathogenic genes of cilia‐related diseases and the methods of targeted therapy are lacking. Many of the same ciliary genes are found in zebra fish, chlamydomonas and other organisms. The highly conserved nature of ciliary genes provided convenience for our study, and it will certainly be interesting to be studied. Our review will provide more references and help for the further research on cilia‐related diseases.

## AUTHOR CONTRIBUTIONS

**Zhanhong Ren:** Conceptualization (equal); writing – review and editing (equal). **Xiaoxiao Mao:** Writing – original draft (equal). **Siqi Wang:** Writing – review and editing (equal). **Xin Wang:** Conceptualization (equal); validation (equal); writing – review and editing (equal).

## FUNDING INFORMATION

This study was supported by the Natural Science Foundation of Hubei Province (Grant No. 2022CFB843), the Xianning Science and Technology Plan Project (Grant No. 2021ZRKX024), Hubei University of Science and Technology School‐level Fund (No. BK202121 and BK202220), Special Project on Diabetes and Angiopathy (No. 2022TNB04), the horizontal scientific research project of Hubei University of Science and Technology (No. 2022HX102 and 2022HX118) and the Scientific Research and Innovation Team of Hubei University of Science and Technology (No. 2022 T01).

## CONFLICT OF INTEREST STATEMENT

The authors confirm that there are no conflicts of interest.

## Data Availability

Data sharing not applicable ‐ no new data generated
